# An Observational Study of Social Interaction Skills and Behaviors in Cornelia de Lange, Fragile X and Rubinstein-Taybi Syndromes

**DOI:** 10.1007/s10803-020-04440-4

**Published:** 2020-03-18

**Authors:** Katherine Ellis, Chris Oliver, Chrysi Stefanidou, Ian Apperly, Jo Moss

**Affiliations:** 1 Cerebra Centre for Neurodevelopmental Disorders, School of Psychology, University of Birmingham, 52 Pritchatts Road, Edgbaston, B15 2TT UK; 2 University of Surrey, Elizabeth Fry Building, Guildford, Surrey, GU2 7XH UK; 3 Present Address: Faculty of Health, Social Care and Education, Anglia Ruskin University, Young Street, Cambridge, CB1 2LZ UK; 4 School of Psychology, University of Birmingham, 52 Pritchatts Road, Edgbaston, B15 2TT UK; 5 Present Address: School of Psychology, University of Surrey, Elizabeth Fry Building, Guildford, Surrey, GU2 7XH UK

**Keywords:** Eye gaze, Fragile X syndrome, Genetics behavioural, Neurodevelopmental disorders, Social behavior

## Abstract

We directly assessed the broader aspects of sociability (social enjoyment, social motivation, social interaction skills and social discomfort) in individuals with Cornelia de Lange (CdLS), fragile X (FXS) and Rubinstein-Taybi syndromes (RTS), and their association with autism characteristics and chronological age in these groups. Individuals with FXS (*p* < 0.01) and RTS (*p* < 0.01) showed poorer quality of eye contact compared to individuals with CdLS. Individuals with FXS showed less person and more object attention than individuals with CdLS (*p* < 0.01). Associations between sociability and autism characteristics and chronological age differed between groups, which may indicate divergence in the development and aetiology of different components of sociability across these groups. Findings indicate that individuals with CdLS, FXS and RTS show unique profiles of sociability.

Individuals with genetic syndromes are at increased risk of social impairment (van Rijn et al. 2014; Galéra et al. 2009; Lesniak-Karpiak et al. 2003; Kasari and Freeman 2001; Wilde et al. 2013). Different syndrome groups show heterogeneity in relation to the nature of strengths and weaknesses across social interaction skills and behaviors, the developmental trajectory of these abilities and the context in which these strengths and weaknesses emerge (Oliver and Woodcock 2008; Karmiloff-Smith 2012).

Sociability is an umbrella term that encompasses a broad range of skills and behaviors that contribute to an individual’s social competence. Most research on the components of sociability within genetic syndromes has focused on autistic traits (Galéra et al. 2009; Mulder et al. 2017; Grados et al. 2017; Moss et al. 2013b; Hogan et al. 2017; Waite et al. 2015; Davenport et al. 2016). Many genetic syndromes have been shown to evidence heightened levels of autistic traits but also show unique profiles of sociability that are not captured fully by diagnostic measures of autism (Moss et al. 2013b, 2016). For example, children with Angelman syndrome often reach clinical cut off scores on assessments of autism (Trillingsgaard and Østergaard 2004; Williams 2010), yet are characterised by high rates of smiling and laugher (Horsler and Oliver 2006), and social approach behaviors (Heald et al. 2013). Identifying autistic characteristics in theses syndromes is essential in ensuring that individual’s receive appropriate services and support (Moss and Howlin 2009). However, other aspects of sociability also warrant investigation in order to tailor this support accordingly (Moss et al. 2013a, b).

In this study, we used, the *Child Sociability Rating Scale* (*CSRS*) (Moss et al. 2013a, b; Oliver et al. 2019), a direct observational behavioral rating scale, to assess the nature and quality of behaviors indicative of social interaction skills, social enjoyment, social motivation and social discomfort in individuals with Cornelia de Lange (CdLS), fragile X (FXS) and Rubinstein-Taybi syndrome (RTS) in order to capture the profile of sociability in these groups.

CdLS, FXS and RTS are neurodevelopmental disorders associated with mild to moderate disability (Oliver et al. 2008; Bennetto and Pennington 2002; Hennekam 2006; Kline et al. 2018), distinguishable by their behavioral phenotypes. Individuals with RTS exhibit greater social competence (Galéra et al. 2009; Hennekam 2006; Moss et al. 2016) in comparison to individuals with CdLS and FXS, whose behavior is characterised by social anxiety (Nelson et al. 2017; Richards et al. 2009; Hall and Venema 2017) and autistic traits (Oliver et al. 2011). Individuals with FXS and CdLS show differences in the profile and developmental trajectory of autism characteristics compared to those with non-syndromic autism (McDuffie et al. 2015; Wolff et al. 2012; Basile et al. 2007; Moss et al. 2008). For example, individuals with CdLS show greater communication difficulties (Moss et al. 2008) compared to non-syndromic autistic individuals, whilst children with FXS show greater impairments in pragmatic language (Martin et al. 2017). These groups show different patterns of change with chronological age. Carer reports indicate that older individuals with CdLS show lower levels of sociability (Moss et al. 2016), lower mood and greater insistence in sameness (Moss et al. 2017) but no changes in autistic traits (Basile et al. 2007; Nakanishi et al. 2012; Cochran et al. 2015). The association between autism and chronological age in FXS has been inconsistent within the literature (Cochran et al. 2015; O'Brien and Bevan 2011; Hatton et al. 2006) and changes with age have been anecdotally reported in individuals with RTS.

We aimed to further characterise the profiles of sociability in individuals with CdLS, FXS and RTS. We first compared components of sociability (social interaction skills, social enjoyment, social motivation and social discomfort) between these groups. We then aimed to refine the descriptions of these profiles by evaluating the pattern of association between sociability and; (1) severity of autism characteristics and (2) chronological age in these syndrome groups. Understanding the association between autism characteristics and other components of sociability will help identify whether and which behaviors may benefit from autism specific interventions and which need to be tailored to syndrome (Moss et al. 2011). Similarly. identifying behaviors that differ across age helps highlight potential unique trajectories and the syndrome specific time points that may be critical for intervention and support.

We hypothesise that:

1. The quality of components of sociability will be higher in individuals with RTS compared to individuals with CdLS and FXS.
2. Individuals with CdLS and FXS will show different profiles of associations between the quality of components of sociability and severity of autism characteristics. No hypotheses can be stated for those with RTS due to the lack of literature investigating autism characteristics, and social interaction skills and behaviors in this group.
3. As chronological age increases in individuals with CdLS, the quality of some components of sociability will decrease. No hypotheses can be stated for those with FXS or RTS due to either mixed or a lack of literature for these groups respectively.

## Methods

### Recruitment

Participants with CdLS, RTS and FXS were recruited via a participant database held by the Cerebra Centre for Neurodevelopmental Disorders at the University of Birmingham and via syndrome support groups. All participants had received a clinical diagnosis of their syndrome by a paediatrician or a clinical geneticist. Participants older than 30 months were required to have a minimum communication and motor age equivalence of 15 months on the Vineland Adaptive Behavior Scales-II (Vineland-II) (Sparrow et al. 2005); participants younger than 30 months were required to have a minimum non-verbal mental age of 12 months to ensure they were eligible to participate in the Autism Diagnostic Observation Schedule, Second Edition (ADOS-2; Lord et al. 2012). Due to reported gender differences in autistic socio-communicative traits in individuals with fragile X full mutation (Hall et al. 2009; Clifford et al. 2007; Hartley et al. 2011; Hessl et al. 2001), only males with FXS were included.

### Measures and Procedure

Individuals were assessed either at the University of Birmingham, at their home and/or at syndrome family support group conferences. The Vineland-II (Sparrow et al. 2005) was administered with the caregiver via telephone.

Cognitive ability was assessed using either the Mullen Scales of Early Learning (Mullen 1995) or the British Ability Scales third edition (BAS3) (Elliott and Smith 2011). Due to a pattern of floor and ceiling effects observed in the expressive language subscales across many participants who took part in the BAS-III, an overall non-verbal mental age was calculated from the mean of participant’s age equivalents on the two non-verbal subscales of the cognitive assessment they participated in to capture participant’s cognitive ability. The ADOS-2 (Lord et al. 2012) was used to assess autism characteristics.

The *Child Sociability Rating Scale* (Moss et al. 2013a, b; Oliver et al. 2019) was used to record the quality and absolute frequency of components of sociability during 10-min intervals across the 30-min ADOS-2 assessment. Behaviors indicative of social enjoyment (positive emotional affect, social responsiveness, negative emotional affect), social interaction (frequency of eye contact, nature of eye contact, social communication style, quality of social communication) social motivation skills (motivation for adult engagement, spontaneous initiation of interaction, focus of attention, frequency of spontaneous physical contact, nature of spontaneous physical contact) and social discomfort (avoidance of social interaction, social anxiety) (see Moss et al. 2013a, b and Oliver et al. 2019 for a full description of items).

### Data Analysis

When data were not normally distributed, non-parametric tests were used. One-way ANOVAs or Kruskal–Wallis tests were used to compare participants’ chronological age, non-verbal mental age and ADOS-2 classification social affect calibrated severity score across syndrome groups. Significant differences were investigated using post-hoc t-tests or Mann Whitney U tests. Chi square tests were used to investigate the proportion of participants who reached the cut-off scores for ASD and autism on the ADOS-2. 2 × 2 chi-squares were used if appropriate to determine which specific groups significantly differ from each other. A *p* < 0.05 cut-off was used to detect differences across groups.

Due to low observed occurrences, the following *CSRS* items were removed from subsequent analyses: *Negative Emotional Affect, Frequency of Spontaneous Physical Contact,* and *Nature of Physical Contact Initiated.* Paired items describing either the frequency or quality of a behavior, such as *Frequency of Eye Contact* and *Nature of Eye Contact*, as well as *Social Communication Style* and *Quality of Social Communication Style *are combined and rescaled to create composite items (*Social Communication Style* and *Social Communication Skills* respectively) scoring between 0 and 4. Combined items were rescaled using the following criteria: 0 = 0, 1–4 = 1, 6–8 = 2, 9–12 = 3, 13–16 = 4.

Mean scores for each item were calculated across the 3 10-min intervals of observation. To account for multiple comparisons, an adjusted *p* value of ≤ 0.01 was used to detect differences across groups in the main analysis, and a *p* value of 0.05 was used for post-hoc analyses.

## Results

### Participants

Thirty-six individuals with CdLS (19 female, M_age_ = 12.42, SD = 10.27, range: 2–50 years), 36 individuals with FXS (0 female, M_age_ = 15.24, SD = 12.59, range: 2–46) and 25 individuals with RTS (13 female, M_age_ = 15.22, SD = 13.78, range: 2–59) were included. No significant group differences were evident for chronological age, non-verbal mental age, or developmental quotient (calculated using participant’s chronological and non-verbal mental ages; see Table 1). CdLS and RTS groups were comparable on gender. Individuals with CdLS and RTS showed significantly lower levels of autism characteristics compared to participants with FXS.

**Table 1:** Participant characteristics for all participants with CdLS, FXS and RTS

	CdLS (*n* = 36)	FXS (*n* = 36)	RTS (*n* = 25)	*p*	Post-hoc tests (*p* < 0.05)
Mean chronological age in years (SD)	12.42 (10.27)	15.24 (12.59)	15.22 (13.78)	0.57	
Gender % female	**53%**	**0%**	**52%**	** < 0.01**	FXS < CdLS, RTS
Non-verbal mental age in years (SD)	3.82 (2.15)^a^	3.45 (1.11)^b^	3.35 (1.35)c	0.79	
Median Developmental Quotient (IQR)	37.87 (29.95)	37.96 (34.79)	27.65 (26.55)	0.44	
ADOS-2 Social Affect CSS (SD)	**4.94 (2.93)**	**6.69 (2.12)**	**5.53 (2.12)**	**0.02**	CdLS, RTS < FXS

Variables with significant differences between syndrome groups are highlighted in bold

^a^Information not available for three participants due to non-completion of the relevant measure

^b^Information not available for three participants due either to floor/ceiling performance (one participant)

^c^Information not available for four participants due either to (1) floor/ceiling performance (one participant) or (2) non-completion of non-verbal scales of a cognitive assessment

### The Broad Profile of Components of Sociability in Individuals with CdLS, FXS and RTS

**Fig. 1:**
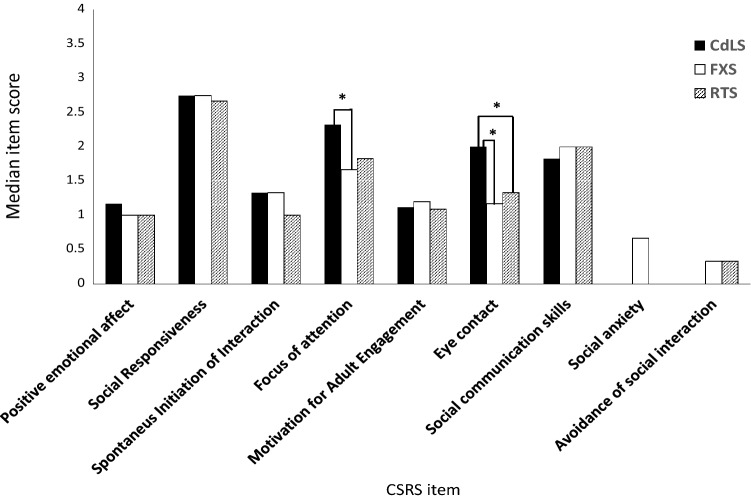
Median item scores on each CSRS item per syndrome

Figure 1 shows the median *CSRS* item scores across all domains in each syndrome. Group differences were found for *Eye Contact* (*Social Interaction Domain*; χ(2) = 16.83, *p* < 0.01; CdLS > FXS, RTS), *Focus of Attention* (*Social Motivation Domain*; χ(2) = 9.22, *p* = 0.01; CdLS > FXS). There were no significant group differences on items in the *Social Enjoyment* or *Social Discomfort* domains.

### Association Between Components of Sociability and Severity of Autism Characteristics in CdLS, FXS and RTS

Table 2 reports correlation coefficients between ADOS-2 *Social Affect* calibrated severity scores (CSS) from the ADOS-2 and mean *CSRS* item scores. A moderate negative association between ADOS-2 *Social Affect* CSS and *Motivation for adult engagement* was identified in CdLS (τ_b_ (34) = − 0.33) A moderate positive association between ADOS-2 *Social Affect* CSS and *Social Anxiety* (τ_b_ (34) = 0.40) was identified in FXS. A moderate negative association between ADOS-2 *Social Affect* CSS and *Eye Contact* (τ_b_ (23) =  − 0.45), and a strong negative association between ADOS-2 *Social Affect* CSS and *positive emotional affect* (τ_b_ (23) =  − 0.60) were identified in the RTS group.

**Table 2:** Kendall Tau correlations for mean CSRS item scores, and ADOS-II Social Affect (SA) CSS and chronological age for each syndrome

*CSRS* item	CdLS		FXS (*p*)		RTS (*p*)	
	ADOS-II SA CSS (*p*)	Chronological age (*p*)	ADOS-II SA CSS (*p*)	Chronological age (*p*)	ADOS-II SA CSS (*p*)	Chronological age (*p*)
Positive emotional affect	0.24 (0.06)	**0.35 (< 0.01)**	− 0.08 (0.56)	− 0.05 (0.70)	− **0.60 (< 0.01)**	− 0.34 (0.02)
Social responsiveness	− 0.31 (0.02)	0.28 (0.02)	0.04 (0.76)	**0.32 (< 0.01)**	− 0.29 (0.06)	0.05 (0.76)
Spontaneous initiation of interaction	0.30 (0.02)	0.07 (0.55)	0.04 (0.76)	0.03 (0.84)	− 0.11 (0.48)	0.04 (0.81)
Focus of attention	− 0.14 (0.26)	**0.34 (< 0.01)**	− 0.09 (0.50)	0.04 (0.75)	− 0.35 (0.03)	0.06 (0.69)
Motivation for adult engagement	− **33 (0.01)**	− 0.04 (0.77)	− 0.09 (0.48)	− 0.18 (0.15)	− 0.16 (0.31)	− 0.06 (0.70)
Eye contact	− 0.24 (0.09)	0.30 (0.03)	− 0.21 (0.12)	− 00.26 (0.04)	− **0.45 (< 0.01)**	− 0.21 (0.12)
Social communication skills	− 0.33 (0.02)	**0.38 (< 0.01)**	0.16 (0.30)	**0.43 (< 0.01)**	− 0.22 (0.21)	0.03 (0.87)
Social anxiety	0.28 (0.04)	**0.40 (< 0.01)**	**0.40 (< 0.01)**	**0.56 (< 0.01)**	0.21 (0.21)	0.12 (0.49)
Avoidance of social interaction	0.15 (0.28)	− **0.34 (0.01)**	0.01 (0.93)	0.03 (0.81)	0.17 (0.31)	0.13 (0.40)

Significant correlations are highlighted in bold

### Association Between Components of Sociability and Chronological Age in CdLS, FXS and RTS

Moderate positive associations between age and *Positive Emotional Affect* (τ_b_ (34) = 0.35), *Focus of Attention* (τ_b_ (34) = 0.34), *Social Communication Skills* (τ_b_ (34) = 0.38) and *Social Anxiety* (τ_b_ (34) = 0.40) were identified in the CdLS group (See Table 2). A moderate negative association between chronological age and *Avoidance of Social Interaction* (τ_b_ (34) = − 0.34) was also found.

In participants with FXS, moderate positive associations between chronological age and *Social Responsiveness* (τ_b_ (34) = 0.32) and *Social Communication Skills* (τ_b_ (34) = 0.43) were evident. Similar to those with CdLS, participants with FXS also showed a moderate positive association between chronological age and *Social Anxiety* (τ_b_ (34) = 0.56). Chronological age was not associated with any of the *CSRS* items in RTS.

As chronological age and non-verbal mental age equivalents are associated with one another in participants with CdLS (τ_b_ (33) = 0.60) and FXS (τ_b_ (32) = 0.54), items that were found to significantly correlate with chronological age were then correlated with participants non-verbal mental age equivalents in participants for which these data were available (Table 1). In participants with CdLS, significant moderate positive associations were found for *positive emotional affect* (τ_b_ (33) = 0.42), *Social Communication Skills* (τ_b_ (33) = 0.55), a strong association for *Social Anxiety* (τ_b_ (33) = 0.60) and a moderate negative association was found for *Avoidance of Social Interaction* (τ_b_ (33) = − 0.36). No association was found between non-verbal mental age and *Focus of Attention* in those with CdLS. In individuals with FXS, moderate positive associations were found between non-verbal mental age and *Social Communication* (τ_b_ (32) = 0.53), and *Social Anxiety* (τ_b_ (32) = 0.48). Pearson’s correlations revealed a positive moderate correlation between participant with FXS’s non-verbal mental age and *Social Responsiveness* (*r* (32) = 0.57).

## Discussion

In this study, observable behaviors indicative of social enjoyment, social motivation, social interaction skills and social discomfort were investigated in individuals with CdLS, FXS and RTS. The first aim was to compare the frequency and quality of these components of sociability between individuals with CdLS, FXS and RTS. Contrary to our hypothesis, behaviors indicative of sociability in RTS were not more frequent or of greater quality to those observed in those with CdLS and FXS, despite previous parent and anecdotal reports of social competence in this group. Rather, those with RTS demonstrated similarities in the quality and frequency of eye contact to those with FXS; a syndrome characterised by gaze aversion (Hall et al. 2009; Cohen et al. 1989). However, findings are consistent with studies that have directly observed operationalised social interaction skills and behaviors indicate subtle difficulties in those with RTS, such as understanding other’s gaze cues and social anxiety, despite showing apparently intact motivation to interact (Powis 2014; Crawford et al. 2019). Other syndromes associated with high levels of sociability such as Down syndrome and Williams syndrome (Moss et al. 2016) have also shown overlap of in specific characteristics of autism on the ADOS-2 (Hepburn et al. 2008; Klein-Tasman et al. 2009; Tordjman et al. 2012), emphasising the importance of direct observations of components of sociability in addition to the use of diagnostic autism measures.

Findings indicate that individuals with FXS and RTS may benefit from interventions aiming to improve the quality and frequency of their eye contact. Individuals with FXS may benefit from desensitisation therapy to help modulate anxiety associated with hyperarousal that is observed in this group, due to impaired neural processing and increased sensitivity when looking at other’s faces (Bruno et al. 2014). Similarly, poor eye contact observed in autistic individuals may be due to abnormally high activation in the subcortical face processing areas when looking at another’s face and gaze (Hadjikhani et al. 2017), suggesting that this may be an overlapping cause of poor eye contact across groups who show gaze aversion.

Individuals with CdLS showed more frequent and appropriate eye contact than individuals with FXS and RTS. Previous literature investigating eye contact in individuals with CdLS has revealed mixed findings (Moss et al. 2012; Sarimski 2007). These mixed findings potentially reflect the genetic heterogeneity and subsequently the variability in the quality of social interaction skills and behaviors previously reported in this syndrome (Gillis et al. 2004; Moss et al. 2017; Nakanishi et al. 2012; Sarimski 2007). Eye contact in those with CdLS is also influenced differently across environments. Whereas individuals with CdLS show more eye contact than those with Down syndrome during a social performance task (Nelson et al. 2017), they also show more fleeting eye contact in comparison to individuals with Cri du Chat syndrome during conditions when the examiner maintained high levels of verbal attention and kept within close proximity to the participant (Richards et al. 2009). These findings highlight that eye contact may vary greatly in those with CdLS dependent upon the social context and type of causal mutation and certain individuals with CdLS may also benefit from intervention aiming to improve eye contact within specific environments.

Individuals with CdLS likely showed more person focused attention than individuals with FXS due to (1) more frequent eye contact with the examiner in individuals with CdLS (indicated by higher scores on the *eye contact* item) and (2) the extreme gaze aversion demonstrated in those with FXS. However, no differences were found in focus of attention between those with RTS or FXS. Despite individuals with RTS showing poor eye contact, their relatively intact social interest (Moss et al. 2016; Galéra et al. 2009; Verhoeven et al. 2010) may have compensated for the effect of poor eye contact and led to more person focused attention in RTS compared to individuals with FXS.

The second aim of the study was to explore the association between the frequency and quality of components of sociability in CdLS, FXS and RTS and the severity of autism characteristics. Different patterns of association were observed between groups. Individuals with CdLS who showed more motivation for adult engagement scored lower on autism characteristics. In FXS, those who showed more social anxiety scored higher on an autism measure. Lower levels of positive emotional affect and reduced quality of eye contact were associated with higher scores on a measure of autism in individuals with RTS.

Whilst it is unsurprising that some components of sociability are associated with the severity of autistic characteristics, it is interesting that the pattern of associations between groups differ. The nature of these association may be mediated by other variables at the neurobiological or cognitive levels that may or may not be the same as those with non-syndromic autism. Both boys with FXS and boys with non-syndromic autism have a heightened likelihood of reaching cut-off scores for social anxiety disorder compared to the typically developing population (Maddox and White 2015). However, direct comparisons reveal differences in the profile of social anxiety in those with FXS and non-syndromic autism, suggesting that the aetiological mechanism driving social anxiety in these groups differ (Scherr et al. 2017). Overall, findings indicate that future work should investigate the refined differences across individuals with syndrome groups and autism on the underlying mechanisms of components of sociability, even when groups show superficial similarities. Outlining these differences may be essential in guiding whether or not individuals with specific genetic syndromes would benefit from autism specific interventions.

The final aim was to explore the association between components of sociability and chronological age in CdLS, FXS and RTS. Older individuals with CdLS showed more frequent and better-quality social communication skills, more positive emotional affect, less avoidance of social interaction *but* more signs of social anxiety. The finding of increased social anxiety in CdLS with chronological age is consistent with other reported areas of change with age including lower levels of sociability (Moss et al. 2016), lower mood and greater insistence in sameness (Moss et al. 2017), and greater impairment in executive function. One suggestion is that the cumulative effects of impaired repair and oxidative stress over time resultant from the syndrome related genetic abnormality (Gimigliano et al. 2012) may account for such changes.

Similarly, older participants with FXS showed more frequent and better social communication skills and social responses and more social anxiety, corresponding to previous reports of higher rates of social phobia (Cordeiro et al. 2011) in older individuals with FXS. This increase in social anxiety with age may be associated with neurobiological changes specific to FXS. Individuals with FXS produce low levels of Fragile X Mental Retardation Protein (FMRP), which regulates expression of proteins involved in synapse formation and function (Tang et al. 2015; Olmos-Serrano et al. 2010) and neural migration (Moro et al. 2006). Subsequently, FMR1 knockout mice show structural deficits in dendritic spines in adulthood but not at 4 weeks old (Kazdoba et al. 2014; Galvez and Greenough 2005) and preliminary evidence has identified Pukinje cell loss or misplacement in the cerebellum in older adults with FXS (Sabaratnam 2000; Greco et al. 2011). Whilst cerebellum damage primarily leads to movement disorders (Choi 2016; Grimaldi 2013), of which older men with FXS are at heightened risk of (Utari et al. 2010), it has also been associated with social anxiety (Caulfield and Servatius 2013; Phillips et al. 2015; Moreno-Rius 2018).

Alternatively, in both CdLS and FXS, the fact that some components of sociability *improve* with chronological age while others demonstrate decline is of interest and suggests that the reported increase in social anxiety may not reflect a general downward trend in behaviors and skills and might be more likely to indicate limited resources to cope with increased cognitive and social demands that individuals face as they become older (Cochran et al. 2019). Interventions for social anxiety may be critical as individuals with CdLS and FXS get older.

Individuals with RTS did not show any associations between age and components of sociability, even on items expected to be associated with cognitive ability (i.e. *Social Communication Skills* and *Social Responsiveness*). The lack of development of these skills in RTS may be due to mutations that act on the cyclic adenosine monophosphate response element binding protein (CREBBP) (Petrij et al. 1995; Park et al. 2014). These mutations have been linked to short- and long-term learning and memory impairments in mice models (Chen et al. 2010) and individuals with RTS show working memory span deficits relative to their overall mental age (Waite et al. 2016). Working memory span is associated with vocabulary acquisition (Newbury et al. 2015; Ellis and Sinclair 1996; Gathercole and Baddeley 1993; Gupta and MacWhinney 1997) and speech and sentence production (Wiseheart and Altmann 2018; Adams 1996; Acheson and MacDonald 2009). These skills are likely to be important for building more complex social communication skills and social responses and may contribute to the lack of development of these components of sociability in those with RTS.

The cross-sectional nature of the study limits the extent to which we can infer causal direction of associations between sociability, autism characteristics and chronological age. Whilst participants took part in both verbal and non-verbal cognitive assessments, non-verbal mental age was the variable that was available for the greatest number of participants. These findings may reflect the uneven profiles of cognitive abilities within these syndromes (Mulder et al. 2017; Grados et al. 2017; Stevens et al. 1990, 2011; Fung et al. 2012; Lorusso et al. 2007). Whilst these data were missing for only a few participants, it is important to take these into account when interpreting the evidence of comparability of level of ability between syndromes included in analyses.

## Conclusions

Profiles of sociability in individuals with CdLS, FXS and RTS show similarities and differences in the broad presentation of components of sociability and in the nature of association with autism characteristics and chronological age. Broad differences were observed, even between groups with similar levels of autistic characteristics. Some components of sociability appear to be associated with autistic traits in those with CdLS, FXS and RTS. Further work is needed to distinguish whether these associations illustrate similarities in underlying aetiology in CdLS, FXS, RTS and iASD, or whether these associations show a unique relationship between autism-like characteristics and broader behaviors. Finally, associations between chronological age and different components of sociability were identified in individuals with CdLS and FXS but not in individuals with RTS. Overall, describing the similarities and differences between profiles of sociability of individuals with CdLS, FXS and RTS has highlighted potential aetiological pathways that drive subtle but important differences in components of sociability across syndromes (Hodapp and Dykens 2001) for future investigation.
